# Serological Evidence and Risk Factors for Swine Influenza Infections among Chinese Swine Workers in Guangdong Province

**DOI:** 10.1371/journal.pone.0128479

**Published:** 2015-05-27

**Authors:** Mengmeng Ma, Benjamin D. Anderson, Tao Wang, Yingan Chen, Dingmei Zhang, Gregory C. Gray, Jiahai Lu

**Affiliations:** 1 Department of Medical Statistics and Epidemiology, School of Public Health, Sun Yat-sen University, Guangzhou, Guangdong Province, China; 2 Infectious Diseases Division, Global Health Institute, Duke University, Durham, North Carolina, United States of America; 3 Zhongshan Center for Disease Control and Prevention, Zhongshan, Guangdong Province, China; 4 Zhongshan Institute, School of Public Health, Sun Yat-sen University, Zhongshan, Guangdong Province, China; 5 Guangzhou Baiyun Center for Disease Control and Prevention, Guangzhou, Guangdong Province, China; 6 Key Laboratory for Tropical Disease Control, Sun Yat-sen University, Ministry of Education, Guangzhou, Guangdong Province, China; College of Veterinary Medicine, CHINA

## Abstract

During July to September 2014, we performed a controlled, cross-sectional, seroepidemiologic study among 203 swine workers and 115 control subjects in Guangdong Province. Sera were tested using a hemagglutination inhibition assay against locally-isolated swine H3N2 and H1N1 viruses and commercially-obtained human influenza viral antigens. We found swine workers had a greater prevalence and odds of seropositivity against the swine H3N2 virus (17.3% vs. 7.0%; adjusted OR, 3.4; 95% CI, 1.1 -10.7). Younger age, self-report of a respiratory illness during the last 12 months, and seropositivity against seasonal H3N2 virus were identified as significant risk factors for seropositivity against swine H3N2 virus. As swine workers in China may be exposed to novel influenza viruses, it seems prudent for China to conduct special surveillance for such viruses among them. It also seems wise to offer such workers seasonal influenza vaccines with a goal to reduce cross-species influenza virus transmission.

## Introduction

Influenza A viruses, with numerous HA-NA subtype combinations of the 18 hemagglutinin (HA) and 11 neuraminidase (NA) genes [1, 2], are recognized to be important zoonotic pathogens, frequently causing infections in humans and a wide range of avian and other mammalian species. Interspecies transmission events have been repeatedly reported by numerous researchers [3–5]. Since pigs are susceptible to influenza viruses of both avian and human origins, their potential role as genetic mixing vessels in the generation of novel pandemic influenza A viruses has long been recognized [6]. Some have thought pigs to have played an important role in the emergence and rapid global dissemination of the 2009 pandemic influenza A (H1N1) virus, a reassortant strain with gene segments from two distinct contemporary swine lineages (North American and Eurasian) [7].

Swine influenza is a common febrile respiratory illness in swine production, causing decreased growth and sometimes mortality among piglets [8]. Based upon case reports and a limited number of epidemiological studies, healthy humans infected with swine influenza virus (SIV) generally develop subclinical or mild respiratory symptoms similar to seasonal influenza. However, occasionally severe morbidity and death may occur, especially among those with underlying medical conditions [9, 10]. Recent cross-sectional seroepidemiologic studies conducted in a number of geographical areas including the USA [11–13], Mexico [14], Germany [15, 16], Thailand [17], and China [18, 19] have suggested that occupational exposure to pigs serves as an important risk factor for SIV infections among man. Thus far three prospective cohort studies [8, 20, 21] have documented incident SIV infections among swine exposed workers with one reassortant swine influenza (H1N1) virus successfully isolated from a sick swine farmer [20]. Though antigenically distinct SIV lineages (subtypes H1N1, H1N2 and H3N2) of North American classical swine (CS), European or Eurasian avian-like swine (EA), triple-reassortant swine (TRIG) and human-like swine orgins were tested in different studies, their findings on the extent to which serologic responses correlate with exposure have been mixed. Besides swine exposure, risk factors associated with elevated antibody titers against SIVs include tobacco use [12, 21] and human influenza vaccination [14, 20]. Two recent studies of Chinese swine workers examined serological evidence of previous infection with avian-like H1N1 SIV virus and identified occupational exposure to pigs as the sole important risk factor [18,19].

In recent decades, China has markedly increased swine production such that the country now produces more than 50% of the world market [22]. New efforts are in progress to further increase pork production by embracing modern Concentrated Animal Feeding Operation (CAFO) production across China. In Southern China, Guangdong Province (capital city Guangzhou) is a geographical area rich in both pork and poultry production, and through the mixing of human and animal species, thought to be a hot-spot for novel zoonotic pathogen generation [19]. To achieve a better understanding of swine influenza ecology in Guangdong Province, we performed a controlled, cross-sectional study to investigate evidence for recent human infections with recently circulating swine H1N1 and H3N2 influenza viruses.

## Materials and Methods

### Participant recruitment and enrollment

**Fig 1:**
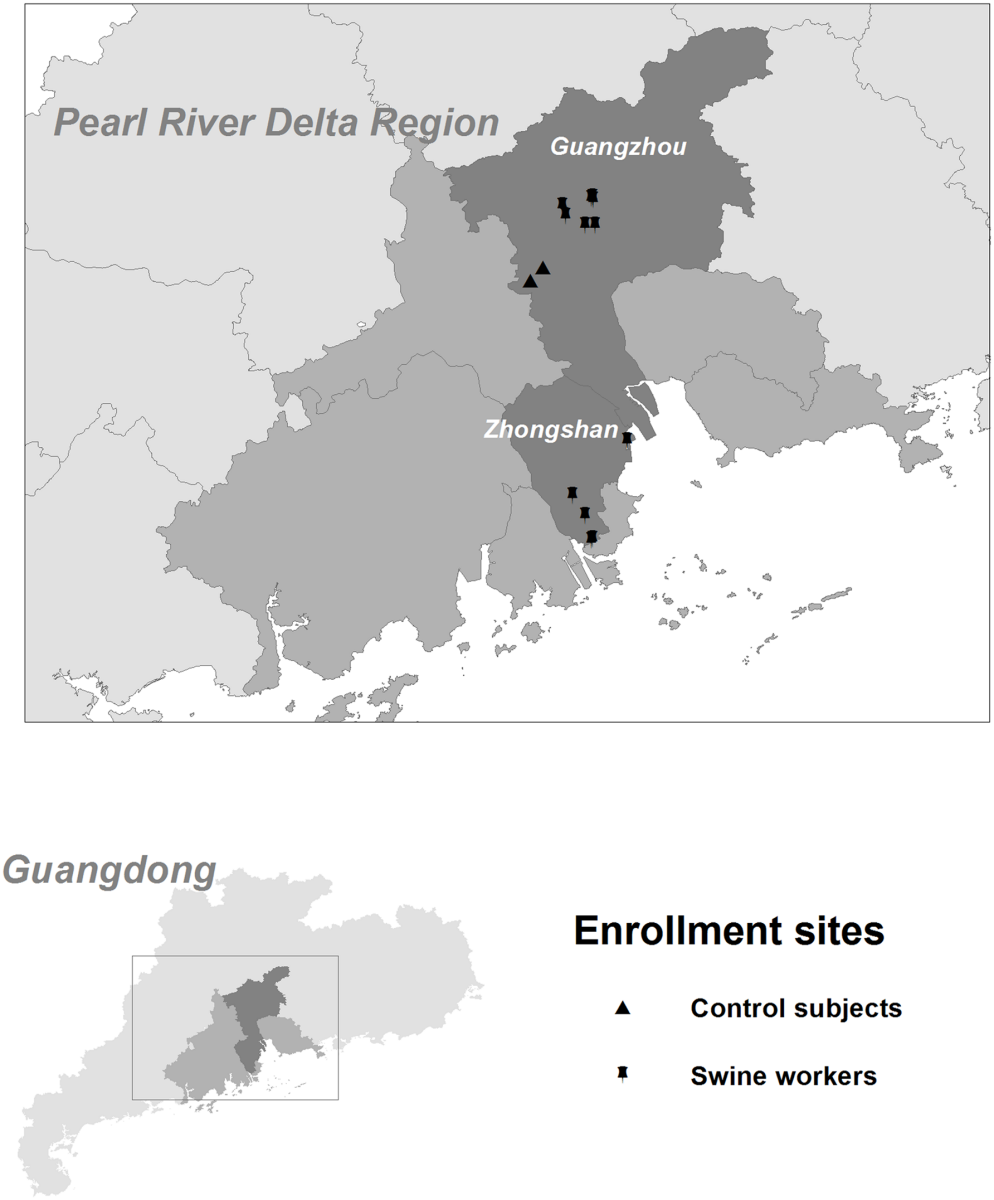
The locations where participants were enrolled in Guangdong Province, China, July-September 2014. Guangdong Province is marked with light gray on the map. The two adjoining cities, Zhongshan and Guangzhou, are highlighted with dark gray while other cities in the Pearl River Delta Region are marked with medium gray. Black triangles indicate enrollment sites for control subjects. For swine workers, enrollment sites are marked with a black pushpin. Note that enrollments were sometimes made at several farms under one pushpin.

This study was reviewed and approved by the institutional review boards at both the School of Public Health, Sun Yat-sen University, China (No. 201331) and the University of Florida, USA (No. 201400417). All participants provided their written informed consent. Study subjects were enrolled from two adjoining cities located in Pearl River Delta region of Guangdong Province (Fig 1): a lightly populated city, Zhongshan (residential population estimated at 3.12 million) and China’s third largest city Guangzhou (residential population estimated 12.7 million) [23]. All swine farms within this area constituted the source population for swine workers in this study.

Study staff contacted swine farm owners and visited swine farms at appointed times through the coordination efforts of local Chinese Centers for Disease Control and Prevention (CDC) and veterinary stations. Through interviewing swine owners and field observation, study workers also completed farm enrollment forms to capture basic farm information, including size, scale, environmental features and management patterns. Swine workers were invited to participate if they self-reported currently living or working at a swine production site for no less than 10 weeks with exposure to pigs or swine manure as part of their daily activity for more than 5 cumulative hours per week. Persons who self-reported immune compromising conditions, acute respiratory tract infections, or pregnancies (for women) at the time of enrollment, were not included in this study.

We enrolled persons ≥18 yrs of age with a goal of enrolling swine workers with intense and prolonged exposure to pigs. After the collection of 5 ml of blood, study participants were interviewed face-to-face and an enrollment questionnaire was completed collecting data on demographics, occupational exposures, community and household exposures, medical and smoking history, vaccination history, and respiratory illness history.

Due to a number of constraints, we combined our study with a community surveillance project contemporarily conducted among urban residents in Guangzhou. We selected two communities (no swine farms located within a 20 kilometer radius) to recruit control subjects. Residents who did not meet the inclusion criteria for swine exposure and who were roughly comparable to swine workers by 10 yr age group and gender, were sought. However, no specific matching was performed. Enrollments were performed at local Community Health Centers from among patients seeking physical examinations. Consenting participants’ blood specimens were collected at the center and their enrollment questionnaires were completed through a later telephone interview by our investigators.

### Laboratory methods

Whole blood specimens (5ml yellow-top tube with inert separation gel at the bottom) were transported on ice to biosafety level 2 laboratories at Zhongshan CDC or Sun Yat-sen University in Guangzhou within 24 hrs after collection. Upon arrival, specimens were accessioned and then centrifuged at 3000 rpm for 15 min to separate serum. Sera were then preserved in 3 aliquots at -80°C until processed. Sera were studied at Guangdong Provincial CDC with hemagglutination inhibition (HI) assay testing.

All serum samples were tested against four antigens of recently circulating swine and human influenza A viruses: A/swine/Guangdong/SS1/2012(H1N1), A/swine/Guangdong/L5/2010(H3N2), A/California/07/2009(H1N1) (pandemic H1N1), and A/Victoria/210/2009(H3N2) (seasonal H3N2). Specifically, the two swine virus strains were isolated from swine farms locally in Guangdong Province by the veterinary laboratory in South China Agriculture University and grown in embryonated chicken eggs. A/swine/Guangdong/SS1/2012(H1N1) is a recently isolated avian-like SIV, genetically related to avian-like H1N1 strains circulating in Europe, Hong Kong and mainland China [19]. A/swine/Guangdong/L5/2010(H3N2) is a double-reassortant SIV of classical H1N1 SIV and recent (2000s) human H3N2 influenza virus lineage [24]. The remaining two human influenza viruses were obtained commercially (Sinovac Biotech, China). In addition, three commercial avian origin influenza virus HA antigens H5, H7, and H9 (Weike Biotech, Harbin Veterinary Research Institute, China) were used to test antibody responses.

We followed HI assay protocols previously documented as recommended by the WHO [25]. Briefly, 1 volume of sera was pretreated at 37°C with 3 volumes of receptor-destroying enzyme (Denka Seiken, Japan) for 18–22 hrs. After being heated at 56°C for 30 min to inactivate the enzyme, sera were then hemadsorbed with chicken blood (for human strains), turkey erythrocytes (for swine strains and avian H9 strains) or horse erythrocytes (for avian H5 and H7 strains). The starting dilution of serum in this study was 1:10 and the largest titer was 1:320. HI titer results were reported as the reciprocal of the highest dilution of serum that inhibited antigen-induced hemagglutination of a 0.75% (from chickens) or 1% (from turkeys) or 1% (from horses) suspension of erythrocytes (corresponding to erythrocytes used for hemadsorption).

### Statistical methods

Questionnaire data were managed using EpiData 3.1 software (http://epidata.dk/). Data were entered twice by two independent persons and verified with structured query language. Questionnaire and laboratory data were later merged into a master dataset as linked by individual study participant numbers. We employed SPSS ver 21.0 (IBM, USA) in the analyses. HI titer results were first studied as dichotomous outcomes (HI titers of ≥1:40 were considered to be evidence of previous infection [11, 13]) using the two-sided Chi-square tests or the Fisher's exact test, with 95% Confidence Intervals (CIs) calculated for Odds Ratios (ORs). Geometric mean titers (GMTs) for HI assays were also calculated for each virus strain and compared with potential risk factors using the Mann-Whitney U rank sum test. In the GMT comparisons, negative titers were assigned the value of half the minimum detectable titer (1:5) and titers greater than the final dilution were assigned the value of twice the largest titer (1:640) [26]. Bivariate analyses were firstly performed to identify independent predictors associated with the dichotomous serologic outcomes. Risk factors with P values ≤0.1 were included in a stepwise, backwards elimination, unconditional logistic regression model [13]. Only risk factors with P values <0.05 were retained in the final model.

## Results

### Participants

From July to September 2014, using informed consent, we enrolled a total of 318 study participants. Among the swine workers, 203 (69.2%) of 293 eligible subjects volunteered to participate. Swine workers [Zhongshan (N = 130) and Guangzhou (N = 73)] were enrolled at 6 large and 6 medium-sized commercial swine CAFOs based on size thresholds (number of pigs confined) developed by the Environmental Protection Agency, USA [27]. The controls (N = 115) were enrolled from two districts of Guangzhou (no swine farms located within a 20 kilometer radius) which were located in between two swine farms areas (Fig 1).

Swine workers were more likely to be male (70.0%) (Table 1) and middle-aged (mean, 40.2 years), with the 40–49 yrs age-group accounting for the largest percentage (35.0%). Participants were asked if they were born outside of Guangdong Province and nearly half of swine workers were migrant workers from other parts of China (46.7% vs. 20.0%, swine workers vs. controls, respectively). More than 70% of swine workers self-reported a primary or lower education level. Economic status, as suggested by monthly food expenditure, was also significantly lower among swine workers when compared to community control subjects. All subjects reported access to indoor water in the household. Of the 203 swine workers enrolled, 90.6% self-reported being farm owners or farm workers and 16.3% reported being veterinarians or veterinary assistants. Occupations for the 115 control subjects were relatively nonspecific: 32.9% were unemployed (retirees, housewives, etc.), 20.3% were business workers, and 41.7% were otherwise engaged such as university students (data not shown). Control subjects (Table 1) were found to have more reports of non-workplace live pigs and poultry exposure, heart disease, hypertension, or stroke, other chronic medical problems such as diabetes, while swine workers were more likely to take medications during the last 30 days and to have developed a respiratory illness during the last 12 months. Specifically, 22 out of 63 medication reports among swine workers were for respiratory illnesses and 14 were for gastrointestinal diseases. Tobacco products use was similar between study groups. Human influenza vaccination was rarely reported by swine workers and or by control subjects (4.8% vs. 3.9%, respectively).

**Table 1:** Demographic and other characteristics of study participants upon enrollment, Guangdong Province, China, July-September 2014.

Characteristic	Total	Swine workers	Control subjects
	(N = 318)	(N = 203)	(N = 115)
**Gender**			
**Male**	218	142 (70.0)	76 (66.1)
**Female**	100	61 (30.0)	39 (33.9)
**Age, years**			
**18–29**	58	38 (18.7)	20 (17.4)
**30–39**	79	51 (25.1)	28 (24.3)
**40–49**	111	71 (35.0)	40 (34.8)
**≥50**	70	43 (21.2)	27 (23.5)
**Age, mean years**	318	40.2	41.2
**City** ^**a**^			
**Zhongshan**	130	130 (64.0)	0 (0.0)
**Guangzhou**	188	73 (36.0)	115 (100.0)
**Born outside Guangdong Province** ^a^ ^,^ ^b^			
**Yes**	106	91 (46.7)	15 (20.0)
**No**	164	104 (53.3)	60 (80.0)
**Highest level of education completed** ^a^ ^,^ ^b^ ^,^ ^c^			
**Primary (Grades 1–6) and lower**	169	141 (70.1)	28 (35.4)
**Secondary and tertiary (Grades 7–12), Technical secondary school**	76	43 (21.4)	33 (41.8)
**2 year College degree and higher**	35	17 (8.5)	18 (22.8)
**Average monthly expenditure for food within the household over than 450 RMB** ^a^ ^,^ ^b^			
**Yes**	215	143 (82.7)	72 (97.3)
**No**	32	30 (17.3)	2 (2.7)
**Non-workplace exposure to live pigs** ^a^ ^,^ ^b^			
**Yes**	24	13 (6.4)	11 (13.9)
**No**	258	190 (93.6)	68 (86.1)
**Non-workplace exposure to poultry** ^a^ ^,^ ^b^			
**Yes**	31	16 (7.9)	15 (19.0)
**No**	251	187 (92.1)	64 (81.0)
**Chronic breathing problems** ^**b**^			
**Yes**	8	7 (3.6)	1 (1.3)
**No**	267	190 (96.4)	77 (98.7)
**Heart disease, hypertension, or stroke** ^a^ ^,^ ^b^			
**Yes**	14	5 (2.6)	9 (11.5)
**No**	257	188 (97.4)	69 (88.5)
**Other chronic medical problems** ^a^ ^,^ ^b^			
**Yes**	10	2 (1.0)	8 (10.4)
**No**	259	190 (99.0)	69 (89.6)
**Ever used tobacco products** ^**b**^			
**Yes**	102	80 (39.8)	22 (28.2)
**No**	177	121 (60.2)	56 (71.8)
**Ever took medications during the last 30 days** ^a^ ^,^ ^b^			
**Yes**	76	63 (31.7)	13 (16.5)
**No**	202	136 (68.3)	66 (83.5)
**Ever received vaccination for human influenza** ^b^			
**Yes**	12	9 (4.8)	3 (3.9)
**No**	253	180 (95.2)	73 (96.1)
**Developed a respiratory illness during the last 12 months** ^a^ ^,^ ^b^			
**Yes**	152	124 (61.4)	28 (35.4)
**No**	129	78 (38.6)	51 (64.6)

Data are no. (%) of subjects, unless otherwise indicated. Two-sided Chi-square tests or Fisher’s Exact Test were used for dichotomous data, and Independent samples T-test was used for continuous data, unless otherwise indicated.

^a^ Significantly different than control subjects at α = 0.05.

^b^ Variable has some missing data.

^c^ Mann-Whitney U rank sum analysis used.

### Seroprevalence

No serum samples were found to be seropositive against any of the three avian influenza viruses (H5, H7, and H9). Swine workers had much greater odds than did control subjects of being seropositive against the swine H3N2 virus (17.3% vs. 7.0%; OR, 2.8; 95% CI, 1.3–6.3) (Table 2). Among the whole population, only one seropositive sample against swine H1N1 virus was found among swine workers (a veterinarian) indicating no statistically important difference compared to control subjects (0.5% vs. 0.0%). Swine workers demonstrated no increased odds of seropositivity against either of the human influenza strains compared to controls: seasonal H3N2 virus (32.7% vs. 32.2%; OR, 1.1; 95% CI, 0.7–1.7) and pandemic H1N1 virus (5.9% vs. 7.8%; OR, 0.7; 95% CI, 0.3–1.8).

**Table 2:** Hemagglutination inhibition serologic assay ^a^ results against swine, and human influenza viruses for participants' sera, Guangdong Province, China, July-September 2014.

Virus	Total	Swine workers	Control subjects	Unadjusted OR
	(N = 317^b^)	(N = 202)	(N = 115)	(95% CI)^c^
**Swine H1N1**				no convergence ^d^
**Positive**	1 (0.3)	1 (0.5)	0 (0.0)	
**Negative**	316 (99.7)	201 (99.5)	115 (100.0)	
**Swine H3N2**				2.8 (1.3–6.3)
**Positive**	43 (13.6)	35 (17.3)	8 (7.0)	
**Negative**	274 (86.4)	167 (82.7)	107 (93.0)	
**Pandemic H1N1**				0.7 (0.3–1.8)
**Positive**	21 (6.6)	12 (5.9)	9 (7.8)	
**Negative**	296 (93.4)	190 (94.1)	106 (92.2)	
**Seasonal H3N2**				1.1 (0.7–1.7)
**Positive**	102 (32.2)	66 (32.7)	36 (32.2)	
**Negative**	215 (67.8)	136 (67.3)	79 (67.8)	

Data are no. (%) of subjects, unless otherwise indicated. Two-sided Chi-square tests or Fisher’s Exact Test were used for dichotomous data analysis.

^a^ Hemagglutination inhibition assay, negative = titer <1:40, positive = titer ≥1:40.

^b^ 317 sera were available from the 318 study participants.

^c^ Unadjusted odds ratio was calculated from bivariate analysis with binary logistic regression method for swine workers versus control subjects.

^d^ Indicates data were too sparse for the model to converge.

Geometric mean antibody titers (GMTs) against swine, human, and avian H9 influenza viruses are shown in Table 3 (data not shown for avian H5 and H7 with all negative titers). The GMT against swine H3N2 was higher for swine workers compared to control subjects (1:10.4 vs. 1:7.8), which was consistent with results in the dichotomous comparison. In addition, statistically significant GMT differences between the two groups were also observed against H1N1 SIV and pandemic H1N1 virus (1:5.3 vs.1:5.0 and 1:6.3 vs.1:7.4, swine workers vs. controls, respectively). Both swine workers and control subjects had similar high GMTs against seasonal H3N2 virus (1:14.8 vs.1:13.6). Only three participants’ specimens were found to have detectable titers against avian H9, one titer of 1:20 and one titer of 1:10 among swine workers, and one titer of 1:10 among control subjects (no statistical difference).

**Table 3:** Geometric mean titers (reciprocal shown) of antibodies against swine, human and avian influenza viruses for participants' sera, Guangdong Province, China, July-September 2014.

Study Population	Geometric mean titer, by virus ^a^				
	Swine	Swine	Pandemic	Seasonal	Avian
	H1N1	H3N2	H1N1	H3N2	H9
**Swine workers**	5.30^b^	10.42^b^	6.27^b^	14.84	5.05
**Control subjects**	5.00	7.76	7.44	13.60	5.03

Data not shown for avian H5 and H7 (all negative).

^a^ For GMTs calculation [26], negative titers were assigned the value of half the minimum detectable titer (1:5) and titers greater than the final dilution were assigned the value of twice the largest titer (1:640).

^b^ The geometric mean titers (GMTs) for swine workers were found to differ from those for control subjects at α = 0.05 by Mann-Whitney U rank sum analysis.

### Multivariate analysis

Risk factors from the entire study populations were examined for their association with elevated HI titers (dichotomous outcome) against the swine H3N2 virus (data not shown). Eight of 26 variables common to both exposed and control groups were screened as possibly (P value ≤0.1) associated with the outcome: age-group, self-report of a respiratory illness during the last 12 months, study population, any non-workplace poultry exposure, city, average monthly expenditure for food within the household over than 450 RMB, elevated HI titers against seasonal H3N2 virus, and elevated HI titers against pandemic H1N1 virus (Table 4). These eight variables were included in the stepwise, backward elimination logistic regression model yielding a final model suggesting that swine workers, persons in the younger age-group, self-report of a respiratory illness during the last 12 months, and elevated antibodies to seasonal H3N2 virus were each statistically important risk factors for elevated titers against swine H3N2 virus. We forced elevated antibodies against pandemic H1N1 virus into the final logistic regression model and found that swine workers still had much greater odds than did control subjects of being seropositive against the swine H3N2 virus (adjusted OR, 3.4; 95% CI, 1.1–10.7). We found no evidence for collinearity or interaction problems between covariates in the final model.

**Table 4:** Risk factors for elevated antibodies against swine H3N2 virus, using unconditional logistic regression modeling, among study participants, Guangdong Province, China, July-September 2014.

Variables	Total No.	No. (%)	Unadjusted OR	Adjusted OR
			(95% CI)	(95% CI)
**Study population**				
**Swine workers**	202	35 (17.3)	2.8 (1.3–6.3)^a^	3.4 (1.1–10.7)^a^
**Control subjects**	115	8 (7.0)	Reference	Reference
**Age, years**				
**> = 50**	70	2 (2.9)	0.0 (0.0–0.2)^a^	0.1 (0.0–0.3)^a^
**40–49**	111	7 (6.3)	0.1 (0.0–0.2)^a^	0.1 (0.0–0.3)^a^
**30–39**	79	10 (12.7)	0.2 (0.1–0.5)^a^	0.2 (0.1–0.5)^a^
**18–29**	57	24 (42.1)	Reference	Reference
**Developed a respiratory illness during the last 12 months** ^**b**^				
**Yes**	151	31 (20.5)	3.0 (1.4–6.5)^a^	3.2 (1.2–8.2)^a^
**No**	128	10 (7.8)	Reference	Reference
**Pandemic H1N1** ^**c**^				
**Positive**	21	8 (38.1)	4.6 (1.8–11.9)^a^	3.5 (0.9–13.1)
**Negative**	296	35 (11.8)	Reference	Reference
**Seasonal H3N2** ^**c**^				
**Positive**	102	29 (28.4)	5.7 (2.9–11.4)^a^	4.8 (2.2–10.8)^a^
**Negative**	215	14 (6.5)	Reference	Reference
**Any non-workplace poultry exposure** ^**b**^				
**Yes**	31	1 (3.2)	0.2 (0.0–1.3)	-
**No**	250	40 (16.0)	Reference	
**City**				
**Zhongshan**	129	23 (17.8)	1.8 (1.0–3.5)	-
**Guangzhou**	188	20 (10.6)	Reference	
**Average monthly expenditure for food within the household over than 450 RMB** ^**b**^				
**Yes**	214	25 (11.7)	0.5 (0.2–1.2)	-
**No**	32	7 (21.9)	Reference	

Data are no. (%) of subjects with elevated antibodies against swine H3N2 virus within levels of each covariate, unless otherwise indicated.

^a^ Significantly different than control subjects at α = 0.05.

^b^ Variable has some missing data.

^c^ Hemagglutination inhibition assay, negative = titer < 1:40, positive = titer ≥ 1:40.

## Discussion

Swine-exposed subjects were enrolled from 12 swine CAFOs in Southern China where they experience frequent and intensive contact with large swine populations. Southern China is thought to be within the epicenter of previous influenza epidemics and pandemics [19]. We compared swine workers and unexposed control subjects’ sera antibody responses against two recently circulating SIVs: avian-like A/swine/Guangdong/SS1/2012(H1N1) and human-like double reassortant A/swine/Guangdong/L5/2010(H3N2). Self-reported potential risk factors for seropositivity were examined.

Similar to previous studies [13, 14], our swine-exposed subjects were chiefly middle-aged males. A large proportion of these swine workers were found to have sparse education and lower incomes compared to controls. Control subjects were more likely to report common chronic disease conditions, which may reflect health seeking behavior and thus enrollment or selection bias. Control subjects also reported more non-workplace exposure to live pigs and poultry. This was especially true among retirees and housewives who frequently visit live animal markets to purchase food. In contrast, swine workers reported seldom visiting live animal markets due to the biosecurity rules for their employment. Interestingly, swine workers self-reported more respiratory illness, taking medications for respiratory illnesses, and having more gastrointestinal diseases, as compared to controls, implying a higher potential of being infected when working in swine confined facilities [28].

Our study is the first to demonstrate that Chinese swine workers were more likely to have elevated antibodies against H3N2 SIV compared to controls in bivariate analysis, geometric mean antibody titer, and multivariate comparisons. Younger age, self-report of a respiratory illness during the last 12 months and elevated antibodies to seasonal H3N2 were identified as other significant risk factors for elevated titers against swine H3N2. Interestingly, remarkably distinct from previous studies [13, 14], elderly people were less likely to have elevated H3N2 SIV HI titers comparing to the youngest age group. Such differences in age-associated serological response have been previously documented [11]. While elderly people may have a higher probability of having experienced more intensive and prolonged influenza virus exposure, their antibody titer response often decreases with age. It is also possible that cross-reacting antibodies against human H3N2 viruses may have protected the older swine workers from bumps in their titers against the double-reassortant A/swine/Guangdong/L5/2010(H3N2) we used in this study. In fact, our swine workers’ high odds (adjusted OR, 4.8; 95% CI, 2.2–10.8) of elevated antibodies to seasonal H3N2 virus, supports our decision and that of similar studies elsewhere [13], to control for such cross-reactivity by adding this covariate, as well as the covariate of antibodies against pandemic H1N1 virus, to the final multivariate model to control for such potential confounding. In addition, self-report of a respiratory illness during the last 12 months was also identified as statistically significant in the model, which suggests that prospective cohort studies with active surveillance are required to better understand the epidemiology of SIV infections.

Various international serologic studies [11–13, 15, 17, 20] have demonstrated swine exposed individuals to have a higher odds of being infected with H1N1 SIV than non-swine exposed controls. However, in our study only one serum sample was identified as seropositive against swine H1N1 virus. This may be explained by an antigen mismatch in that we used an avian-like A/swine/Guangdong/SS1/2012(H1N1) virus in our study and perhaps that virus was not seen by the participants’ immune systems. We chose this strain because in recent years two 3-year-old patients have been reported to be infected with this lineage in central and eastern provinces [29]. Other studies in southern and eastern China [18, 19] have similarly found low seroprevalences (11.2% and 10.7%, respectively) of antibodies against avian-like H1N1 SIV.

According to the farm data, all of the 12 farms enrolled adjoin ponds or lakes which serve as habitats for various avian species. Birds control measures were seldom used in the study farms. In fact, one swine farm kept hundreds of ducks and chickens nearby to feed workers and another farm similarly maintained an adjacent pigeon production facility. Though physical segregation was set up, contacts between species were frequently observed by study workers. These factors may contribute to influenza transmission between avian and swine species. Fortunately, among our study subjects, sparse evidence of previous infection with avian H5, H7, and H9 viruses was found. We acknowledge our study was relatively small and not representative of the entire province’s swine worker population as other studies have found much higher prevalences of elevated antibody against avian viruses [30, 31]. It is also possible that our low prevalence may have been due to a mismatch between antigens of avian influenza viruses circulating and those used in our assays.

Our study data support the need to develop targeted surveillance and interventions for populations which are routinely and intensively exposed to swine. It seems quite possible that relatively healthy swine workers may serve as a bridging population to move swine viruses to families and villages as to move human viruses to the pigs they care for [32]. New research suggests that humans may more often transmit human influenza viruses to pigs than they are infected by SIVs from contact with pigs [33]. Vaccination is currently the best strategy to build immunity among swine workers and in so doing, to reduce the risk of cross-species influenza virus transmission and possible novel virus emergence. Disappointingly, the prevalence of influenza vaccination rate (4.5% of the whole study population) reported in our study population was far below levels reported in previous studies for developed countries [13, 14]. Since human influenza vaccine is not covered by China’s Expanded Program on Immunization and swine workers must seek vaccine and pay for it, their vaccination rates are understandingly low (overall mean vaccination rate 9.0%) and influenced by these and other independent factors [34]. Though human influenza vaccination was not identified as a predictor for SIV infections in our study, a strong cross-reactivity between seasonal H3N2 and swine H3N2 viruses has been suggested in our analysis. A study in Mexico also revealed vaccination to be a protective factor [OR 0.05, 95% (CI) 0.01–0.52] in preventing human H3N2 SIV infections [14]. In addition to worker vaccination, it’s also seems imperative to consider swine workers for active influenza surveillance and to better study the swine farm environment such that other interventions might be developed to change high risk environments or behaviors.

Our study had a number of limitations. First, we may have introduced some selection bias in selecting all control subjects from among urban Guangzhou residents and comparing them with rural swine workers. Potential educational, economic and environmental (e.g. air, living conditions) differences between Guangzhou and Zhongshan, or between urban and rural areas may have influenced results. However, in our risk factor analyses, we failed to find evidence that educational or economic levels were important predictors for evidence of SIV infection. Additionally, a sub-analysis among only Guangzhou study subjects (data not shown) identified the same risk factors as the analysis conducted among the whole population, although the swine workers’ OR value was increased from 2.6 [95% (CI) 1.0–6.8, P value = 0.04] to 3.9 [95% (CI) 0.9–16.1, P value = 0.06]. Still, other unknown selection bias may exist without data collected on related variables. In addition, given large number of backyard farms are mixed together with relatively less commercial farms in Guangdong Province, and the local government encourages consolidation between farms, the number of swine farms and workers in the study area is not available. The representativeness of our study subjects could be another source of selection bias. Second, the differences in data collection between swine workers (face-to-face interview) and control subjects (telephone interview) may have led to reporting biases. Third, our questionnaire only collected self-reported information, which is subject to recall bias. Moreover, as previously described, we may have missed important occupational exposure associations by using avian virus antigens that were distinct from avian viruses actually circulating in Guangdong as well as less sensitive HI assay compared to the microneutralization assay as recommended by the WHO for avian influenza viruses. Lastly, our cross-sectional study design would by nature miss incident SIV infections. Further studies should employ a prospective study design to capture more complete SIV infection data and better identify risk factors.

In summary, considering the risk these swine workers have in serving as a bridging population to move SIVs to the general Chinese population [35, 36], and the variety of swine and avian-like SIVs that have been reported to circulate in Chinese pigs, our risk factor data are cause for alarm. It seems prudent that Chinese governmental officials should consider priority of vaccine access for swine workers as well as including them in active surveillance programs for novel influenza virus infections.
